# Sulphate of Quinine in Enlargement of the Spleen, and in Dropsies after Agues

**Published:** 1841-04

**Authors:** 


*Sulphate of Quinine in Enlargement of the Spleen, and in Dropsies after Agues.*—Dr. Levy, physician of the Military Hospital of Val-de-Grace at Paris, has very satisfactorily shown that the dropsical effusions, which not unfrequently supervene upon long neglected agues, are most successfully treated with quinine. He alludes to the researches of MM. Bally, Nonat, Piorry, and other contemporaneous physicians, which have clearly established the superior efficacy of this remedy—to be associated in most cases with the application of the cupping instruments over the left hypocondrium—in dispersing the enlargements of the spleen which so very generally, nay almost always, accompany old intermittent fevers. Indeed, he regards this, the administration of quinine in such cases, as one of the most valuable therapeutic discoveries of recent times. Now, as the dropsical effusions are almost invariably connected with an *infarcted*—to use an old word—state of the spleen, it is a rational deduction to anticipate that the remedy, which is so decidedly efficacious against the *cause*, may not be without its influence upon the *effect*. Certain it is that the use of the ordinarily-resorted-to means, such as diuretics and purgatives, seldom succeeds in dissipating the dropsies which we are at present considering, whereas they may be often dispersed by quinine in full doses.—*Medico-Chirur. Rev., from Gaz. Medicale.*

It is rather odd that this should come to us in the guise of a new discovery. Intermittent fever is rather a rare disease in the north of France, and it is not surprising that many points connected with it should from time to time present themselves with an air of apparent novelty; to us, however, the subject is a familiar one. The engorgements of the spleen are removeable by quinine, not from any specific action of the remedy on the local affection, but because it cures the general disease on which the enlargement depends. Hence, in most cases, topical remedies, such as cups, if the local disorder is at all inflammatory, or blisters, if chronic, add very much to the good effects of quinine.

In fact, intermittent fever is not cured when the paroxysm is for a time arrested; as long as the diseased condition of the blood continues, the mischief still persists. The best test of the continuance of the disease is the color of the skin, as indicative of an anemic state of the economy; this is not infallible, but is a tolerably sure guide. As an adjunct to the quinine, we often prescribe the freshly prepared carbonate of iron in doses of ten to twenty grains in the day. Mercurials are, on the whole, productive of much more mischief than good in this state of things, although, in the diseases of the liver dependant upon intermittent, they are often the best remedy. But, even in the latter case, they may be abused, and increase the chronic engorgement of the liver, which they are designed to correct. There is no certain test of their action, judging a priori, except that they are not suited to those cases in which the blood of the patient is deficient in red globules; they act more certainly when there is more positive evidence of inflammatory action.—*Med. Examiner, Jan.* 23.

